# Supplementary material for: Bio-inspired counter-current multiplier for enrichment of solutes
Source: Nat Commun. 2018 Feb 21;9:736. doi: 10.1038/s41467-018-03052-y (PMC5821707; doi:10.1038/s41467-018-03052-y)
Supplement: Supplementary file 1 — Supplementary Information [file 41467_2018_3052_MOESM1_ESM.pdf]

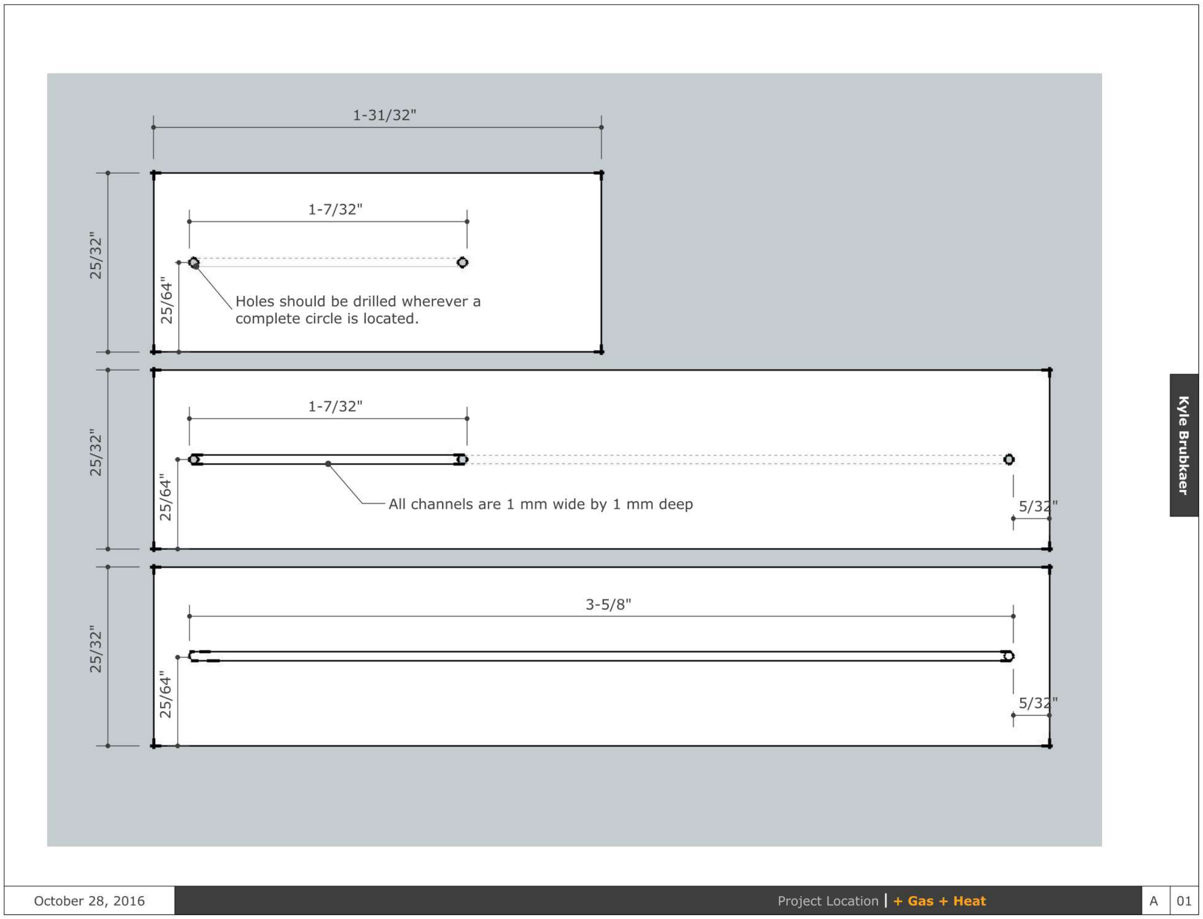


Supplementary Figure 1: Layout of the + gas + Heat or - Gas + Heat variant of the countercurrent amplifier. All dimensions in inches unless noted otherwise.

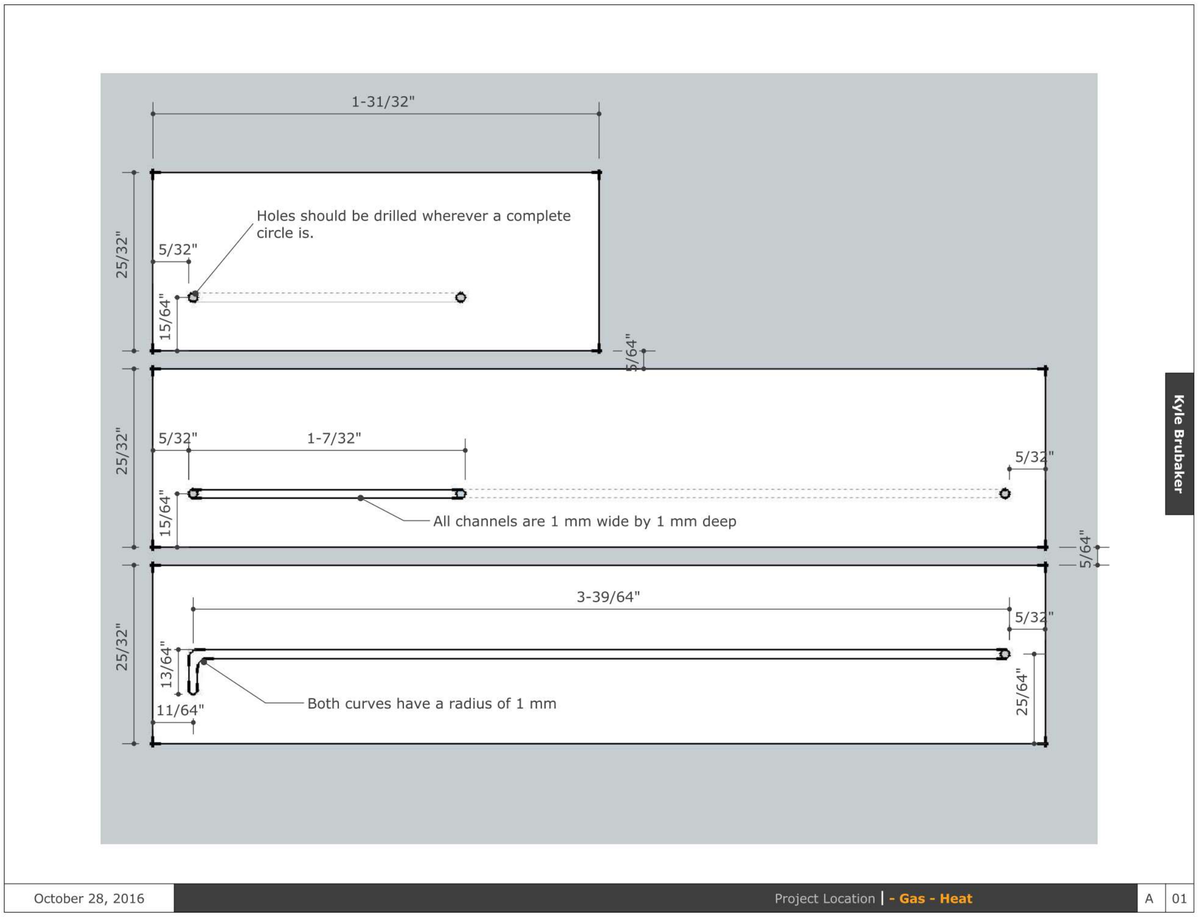


Supplementary Figure 2: Layout of the - gas - heat variant of the countercurrent amplifier. All dimensions in inches unless noted otherwise.

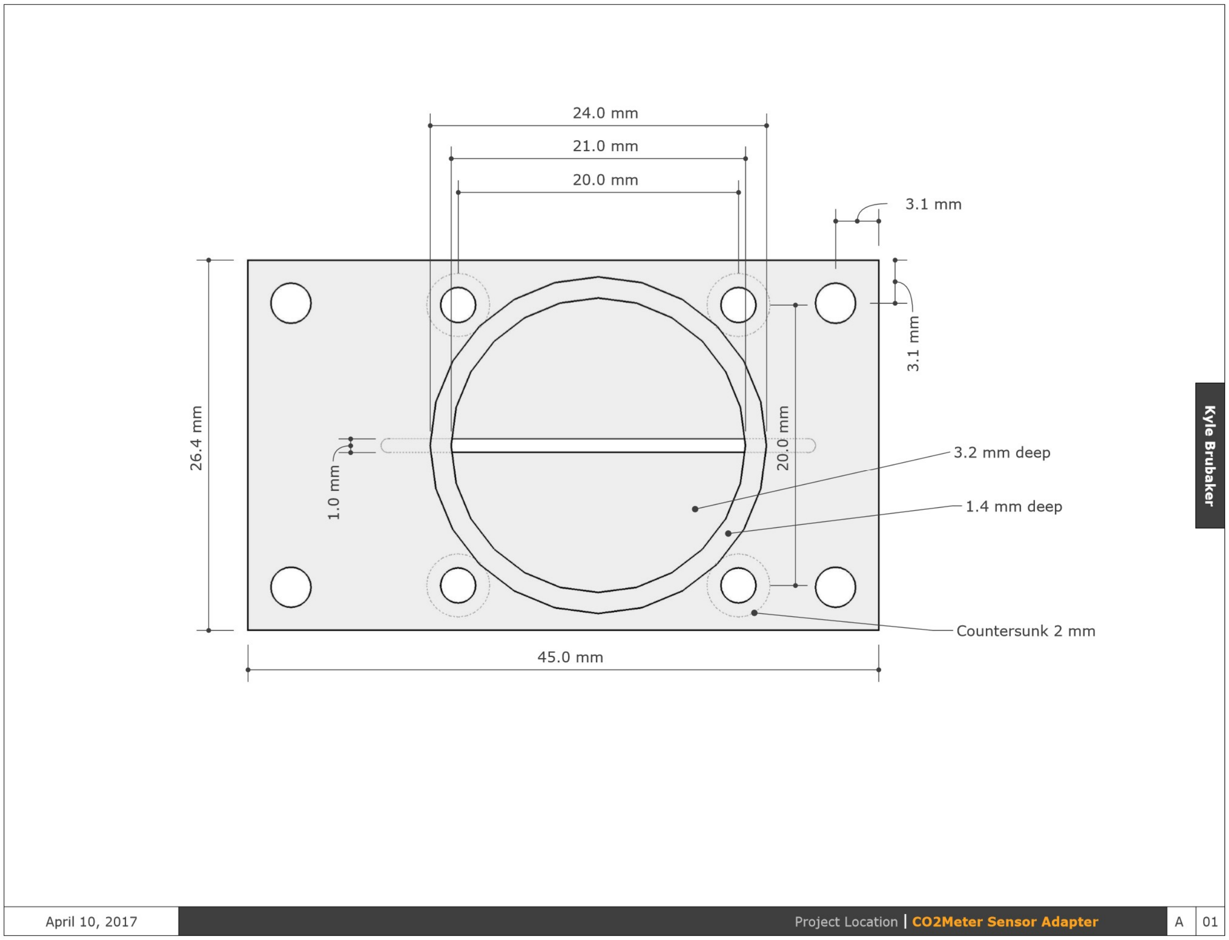


Supplementary Figure 3: Adapter to mount CO2Meter.com sensor directly on the release zone.

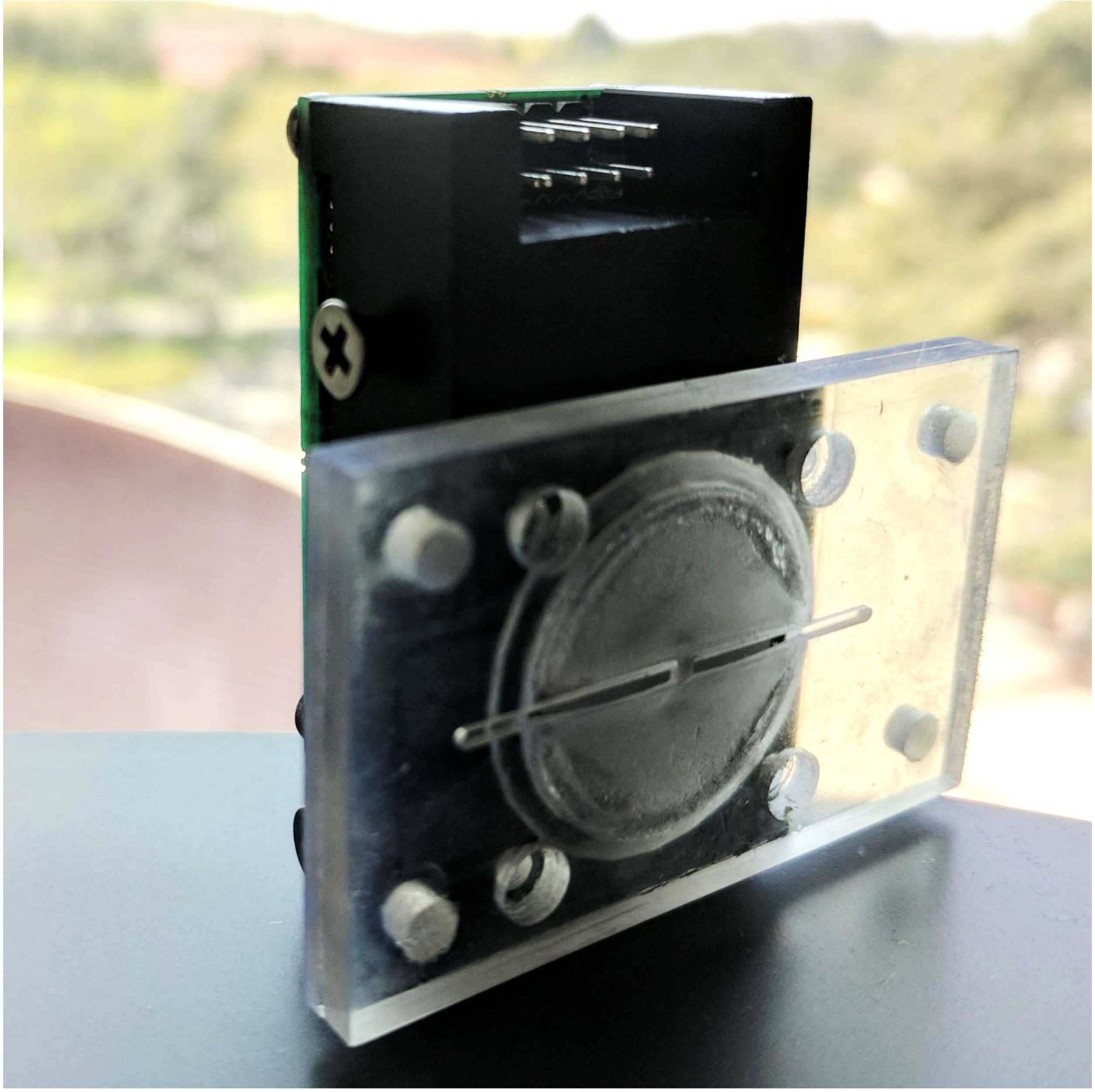

Supplementary Figure 4: Sensor adapter with $CO_2$ sensor in place.

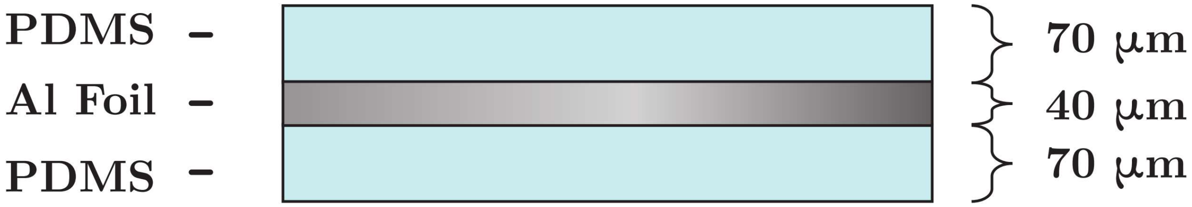


Supplementary Figure 5: Illustration of the PDMS/Al hybrid membrane sandwich

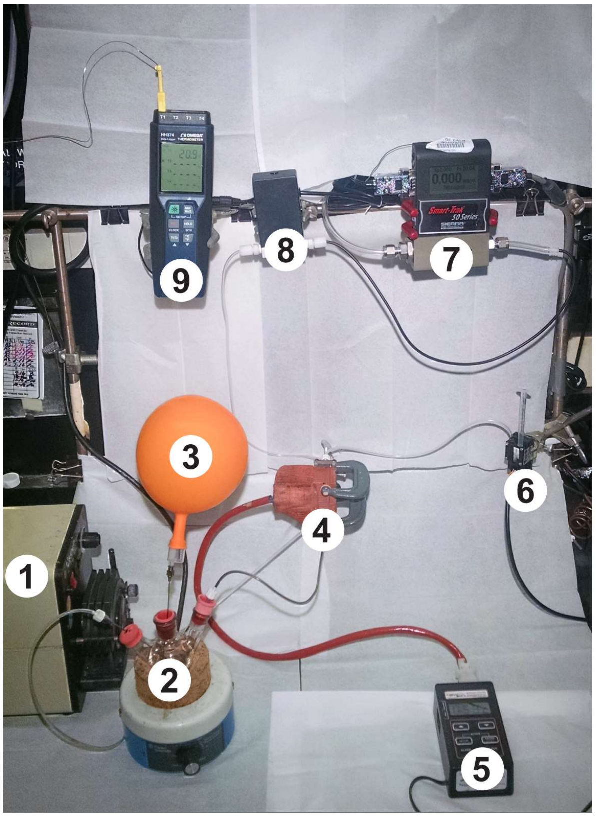


Supplementary Figure 6: Experimental setup of the countercurrent multiplier. (1) Buchler peristaltic pump (2) Capture fluid reservoir (3) Gas balloon (4) Countercurrent amplifier + heating pad (5) New Era syringe heater controller (6) CO2Meter.com GC-0015 $CO_2$ meter (7) Smart-Trak 50 series mass flow controller (8) Cole-Parmer 0-20 ml/min mass flow logger (9) Omega HH374 Thermometer Data Logger.

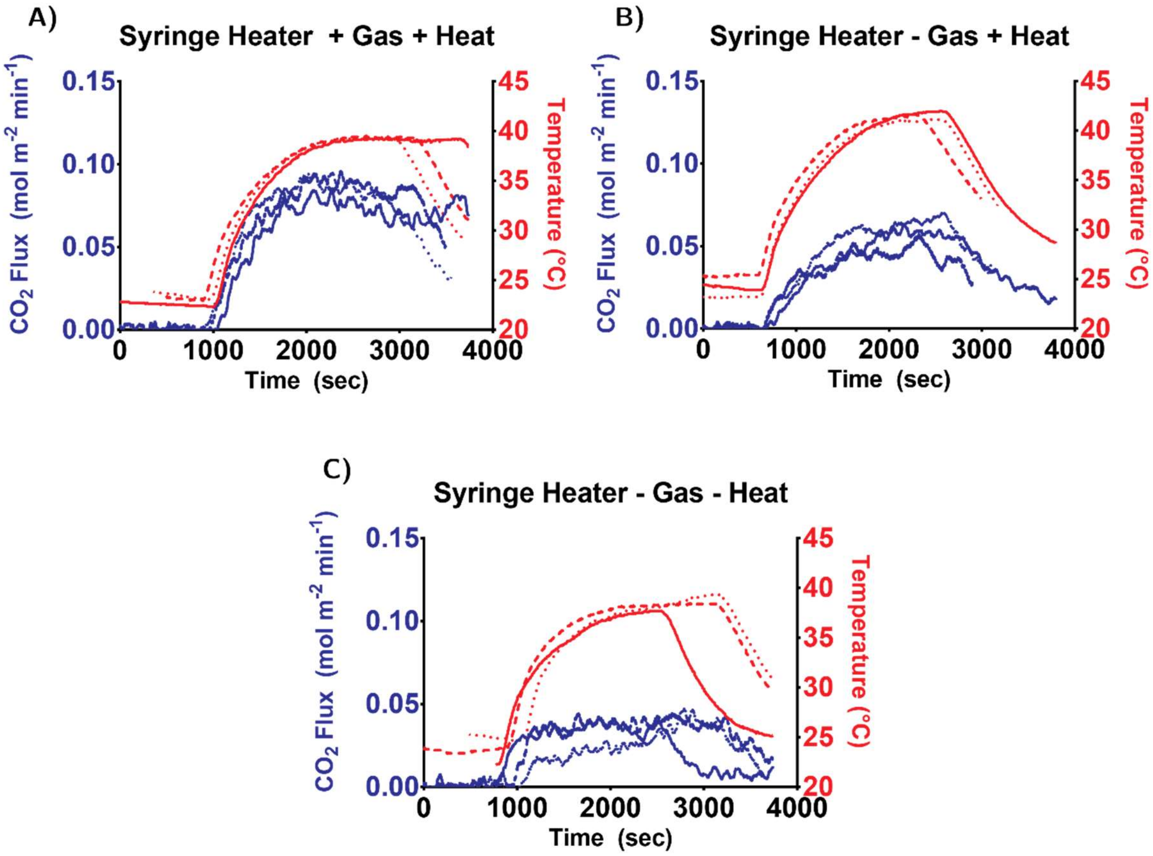


Supplementary Figure 7: Flow data for the $CO_2$/MEA system. Blue curves correspond to the gas flow rates. Red curves correspond to the temperature of the system. A) + Gas + Heat, full countercurrent. B) - Gas + Heat, only countercurrent heat. C) - Gas - Heat, no countercurrent flow.

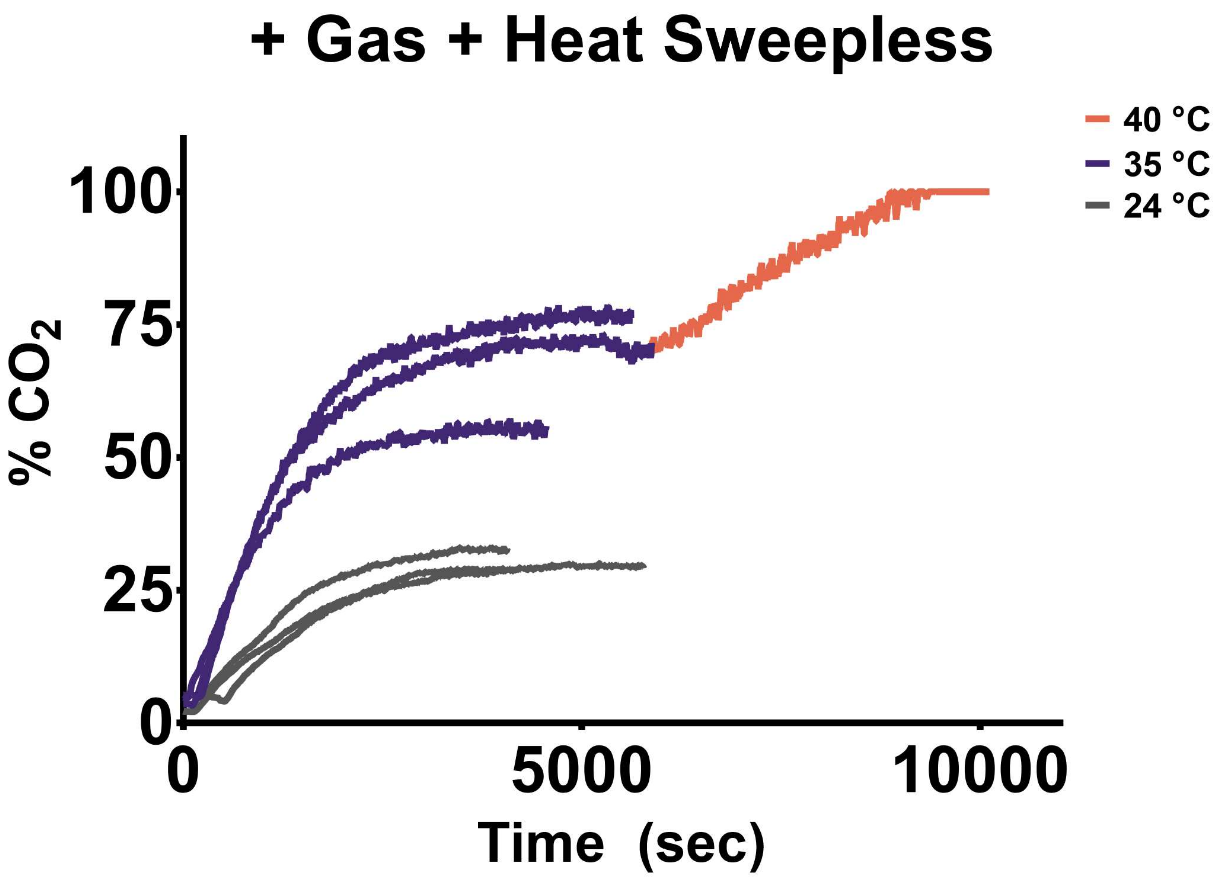


Supplementary Figure 8: Absolute concentration data for measured internal temperatures of 24 °C, 35 °C, and 40 °C. The lower 24 °C trace is composed of two experimental runs. The red 40 °C trace is a continuation of the middle 35 °C trace. Data was obtained using the sensor setup pictured in Supplementary Figure 4.

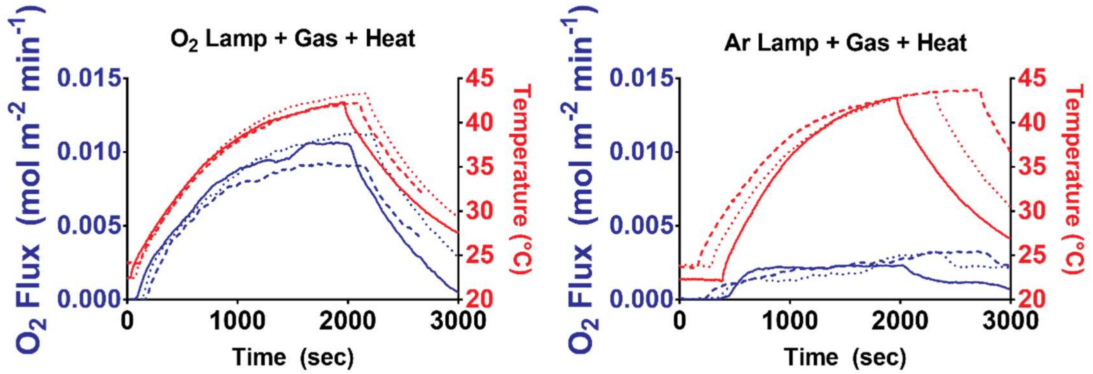


Supplementary Figure 9: Oxygen flow data for the PFO system. To verify that the measured oxygen was not simply from atmospheric oxygen diffusing through the tubing used to transfer capture or sweep gas to the device, we bubbled either oxygen or argon in the perfluorooctane reservoir. Any oxygen seen when argon was bubbled should be the result of atmospheric leakage into the system. Blue curves

correspond to the gas flow rates. Red curves correspond to the temperature of the system. Left: PFO saturated with $O_2$. Right: PFO saturated with Ar.

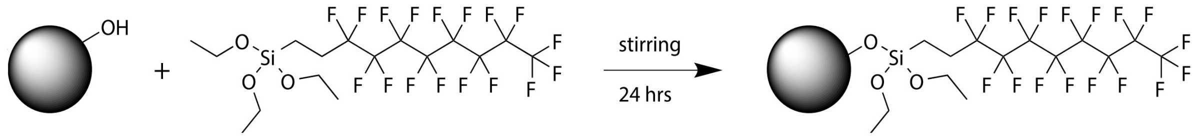


Supplementary Figure 10: Reaction scheme for the silanol coupling of carbon black and 1H,2H,2H,-pefluorodecyltrimethoxysilane.

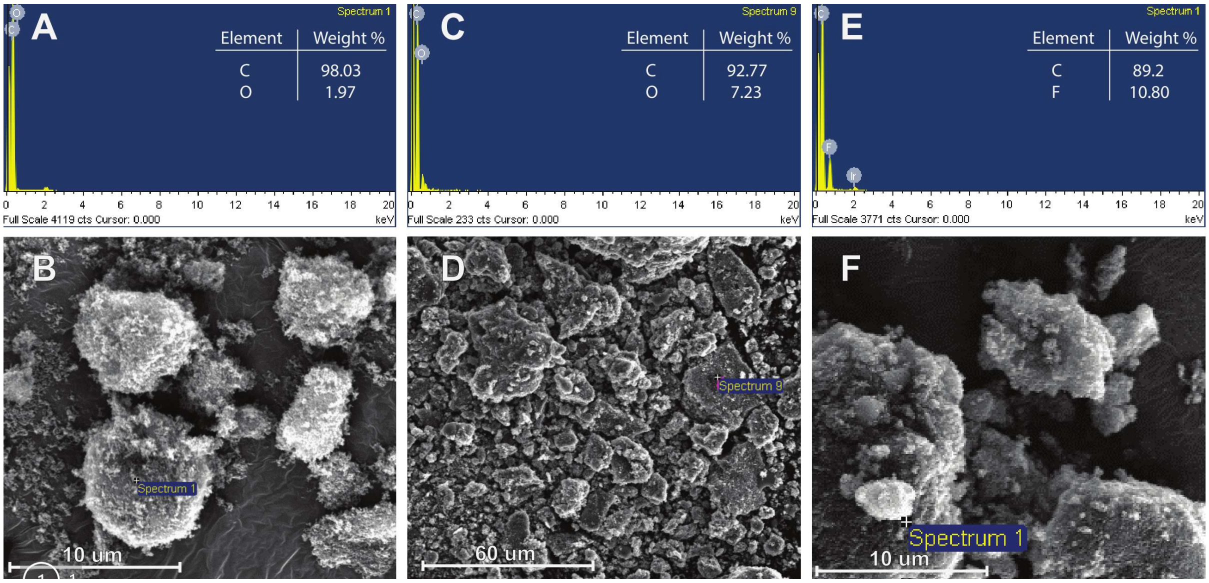


Supplementary Figure 11: EDS characterization data for unmodified carbon black, oxidized carbon black, and perfluorocarbon modified carbon black. All samples sputter coated with iridium. A) EDS spectra for unmodified carbon black. B) SEM image of unmodified carbon black. C) EDS spectra for oxidized carbon black. D) SEM image of oxidized carbon black. E) EDS spectra for 1H,2H,2H,-pefluorodecyltrimethoxysilane modified carbon black. F) SEM image of 1H,2H,2H,-pefluorodecyltrimethoxysilane modified carbon black.

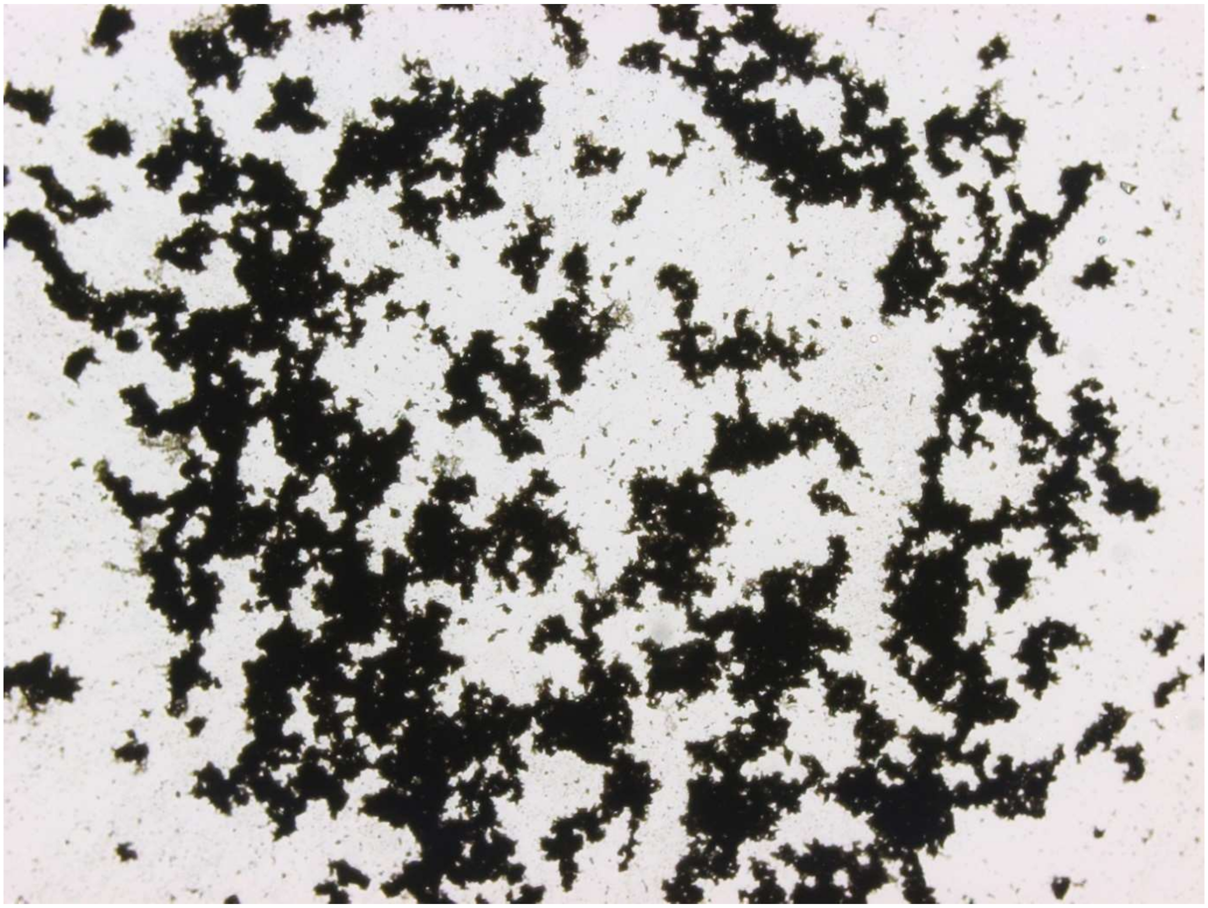

Supplementary Figure 12: Raw carbon black (0.113 % w/w) in PFO after 180 sec. of sonication.

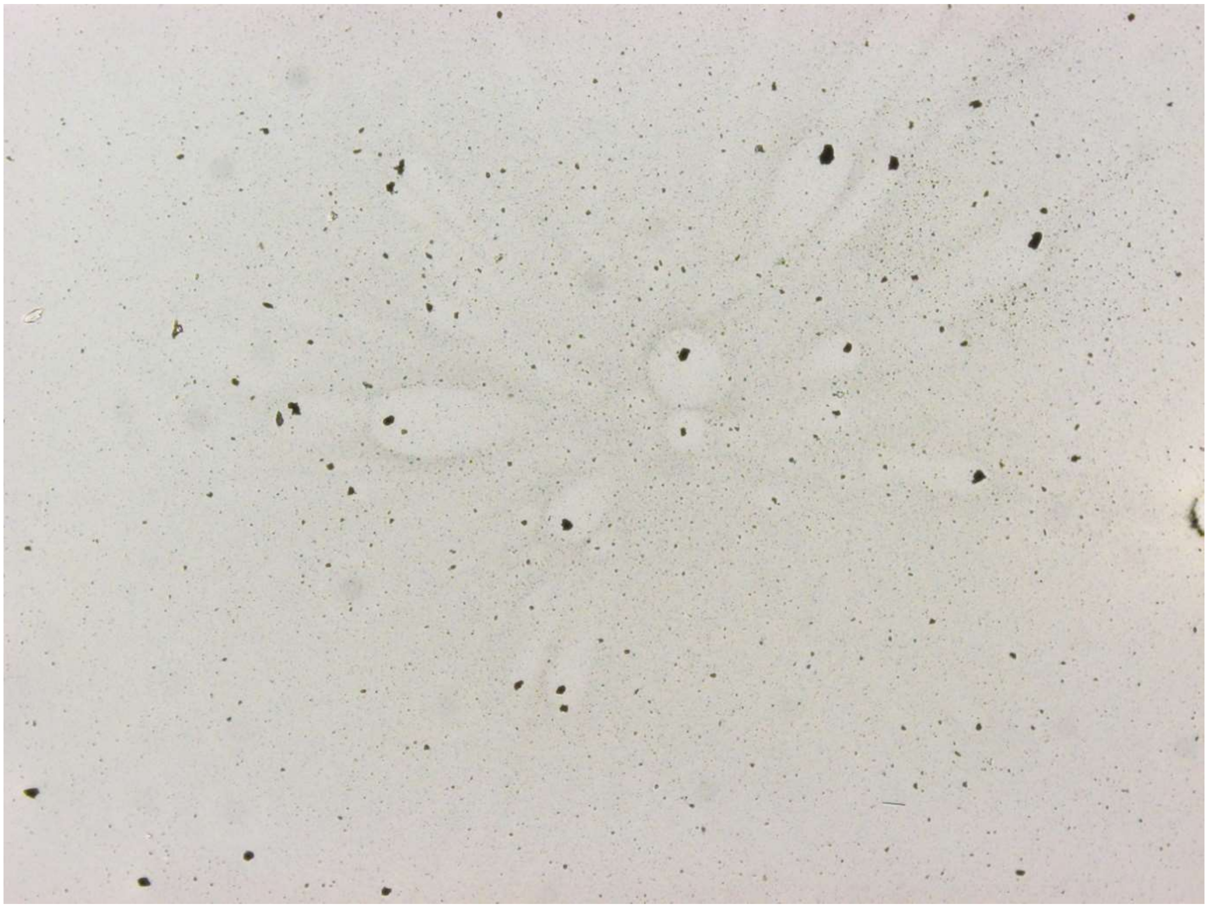

Supplementary Figure 13: Perfluorinated carbon black (0.113 % w/w) in PFO after 180 sec. of sonication.

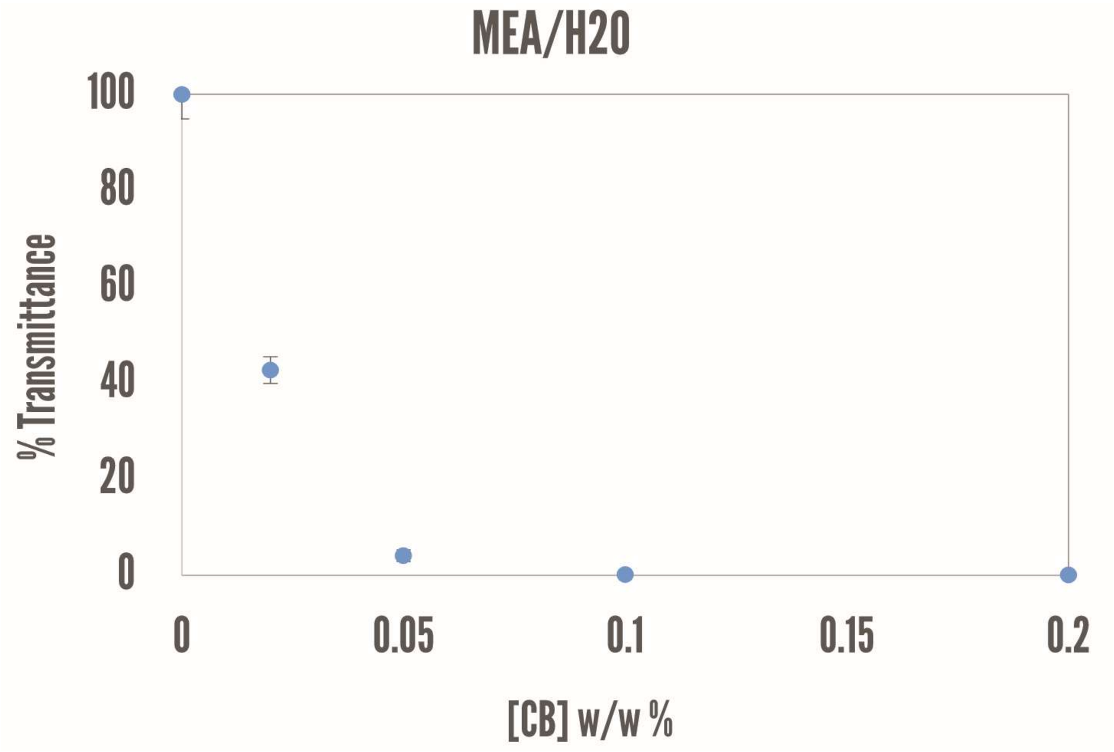


Supplementary Figure 14: Percent transmittance of carbon black nanoparticles in MEA/$H_2O$ with 1 w/w % gum Arabic. A path length of 2 mm was used.

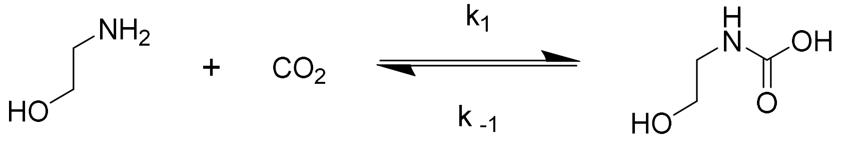


Supplementary Figure 15: Reaction of MEA with $CO_2$. Forward reaction rate is denoted by $k_1$ and the reverse reaction rate is denoted by $k_{-1}$

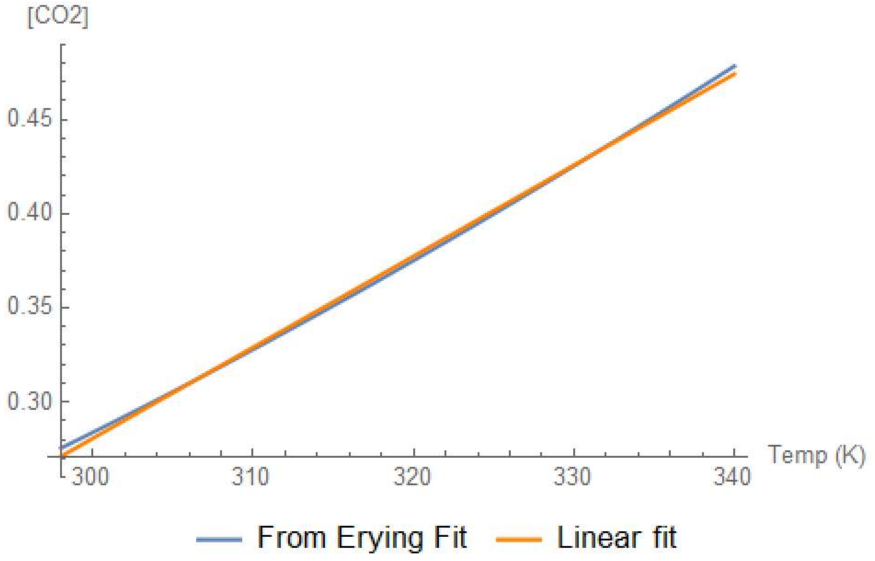


Supplementary Figure 16: Comparison of calculated [CO2] derived from the Eyring fit given by Conway et. al and a linear fit from 298 K to 340 K.

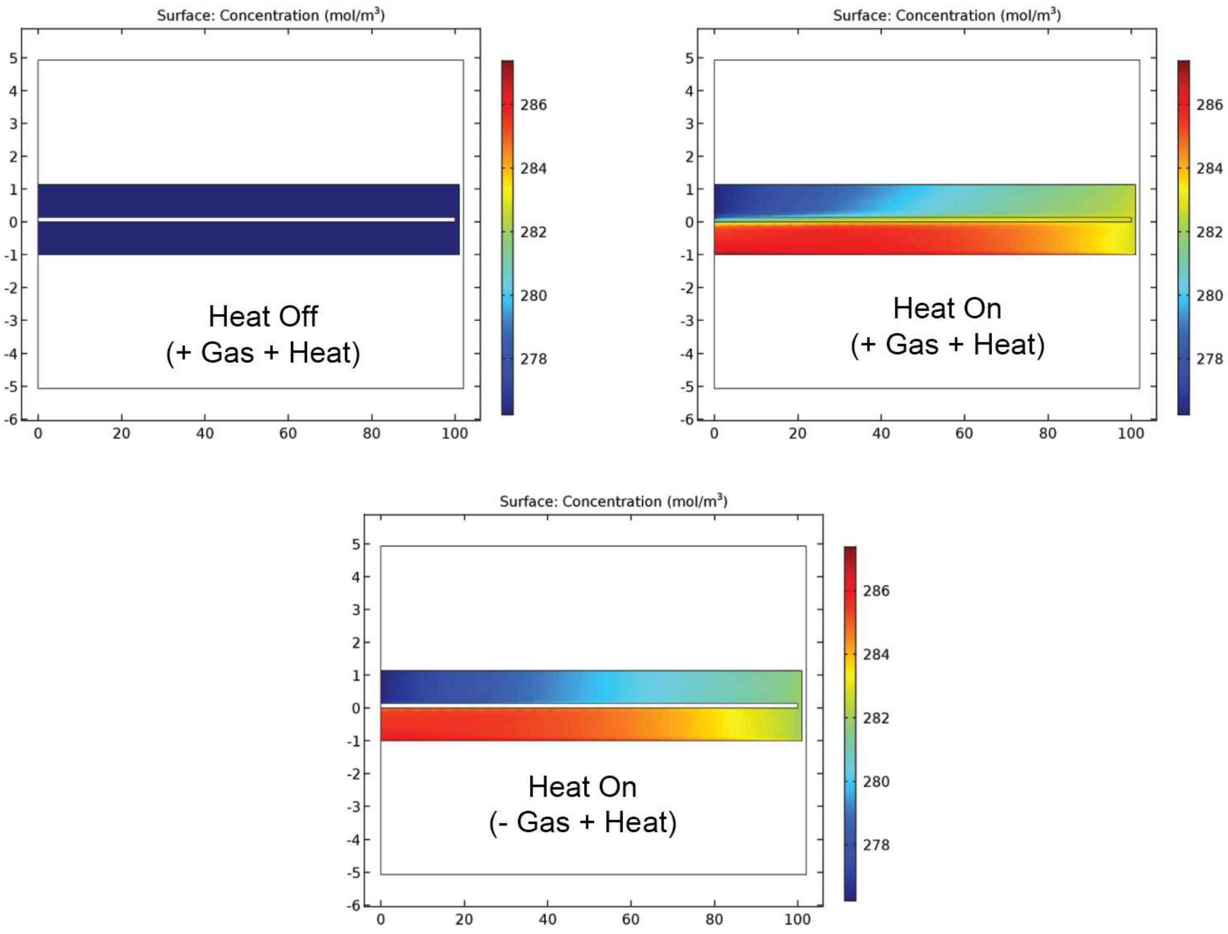


Supplementary Figure 17: Concentration of $CO_2$ in the middle of the inlet and outlet channels with different configurations. Heat is applied from the bottom face of each graphic. Capture fluid flows into the top channel and out through the bottom channel.

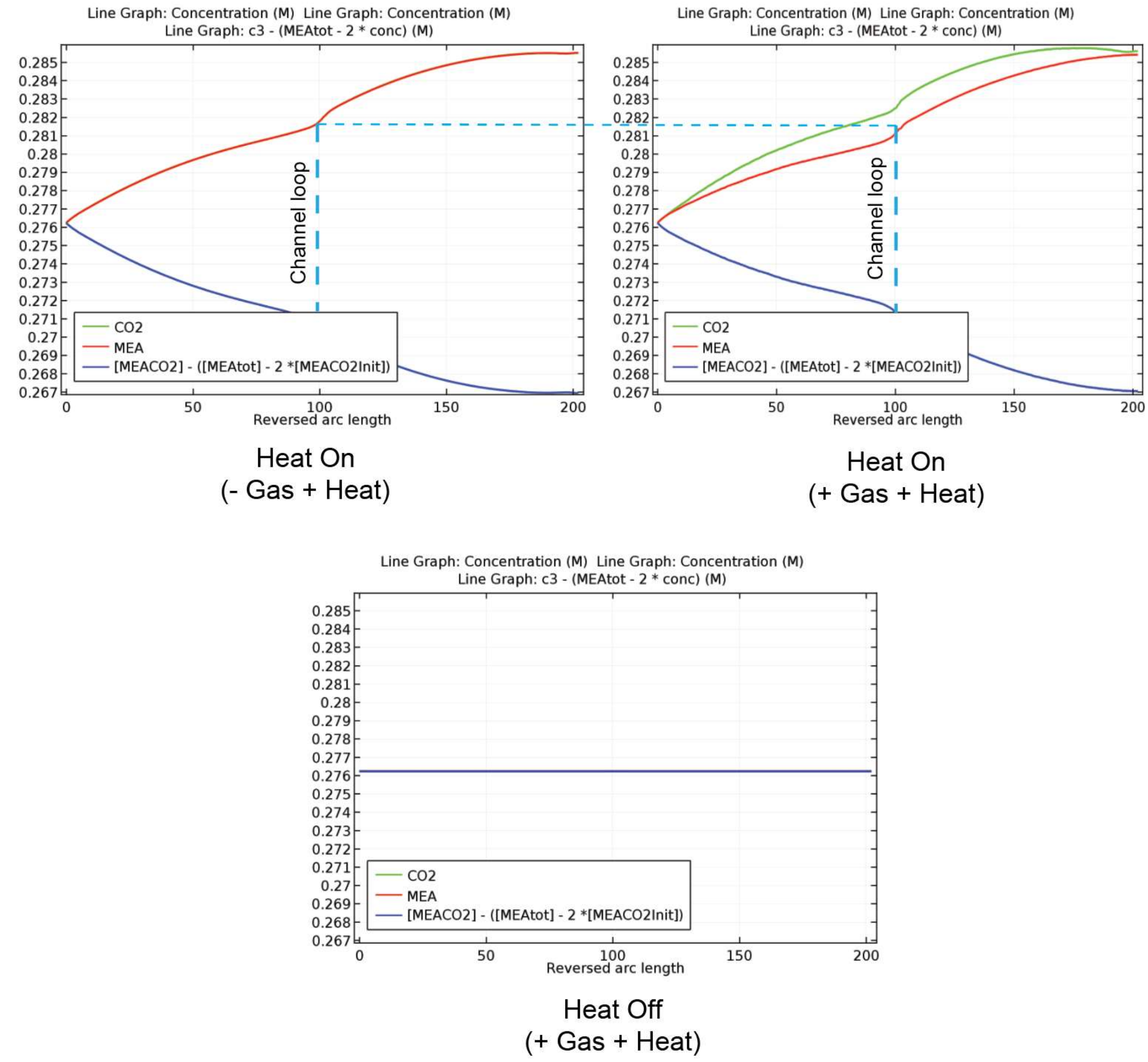


Supplementary Figure 18: Component concentrations in the middle of the inlet and outlet channels. Trigger off (bottom) and trigger on (top). Gas diffusion between channels disabled (left), gas diffusion enabled (right). The concentration of MEA−$CO_2$ was graphed as ([MEA−$CO_2$] − $[MEA]_t - 2 * [MEA]_e$) where $[MEA]_t$ is the total concentration of MEA species in solution and $[MEA]_e$ is the equilibrium MEA concentration without applied heat.

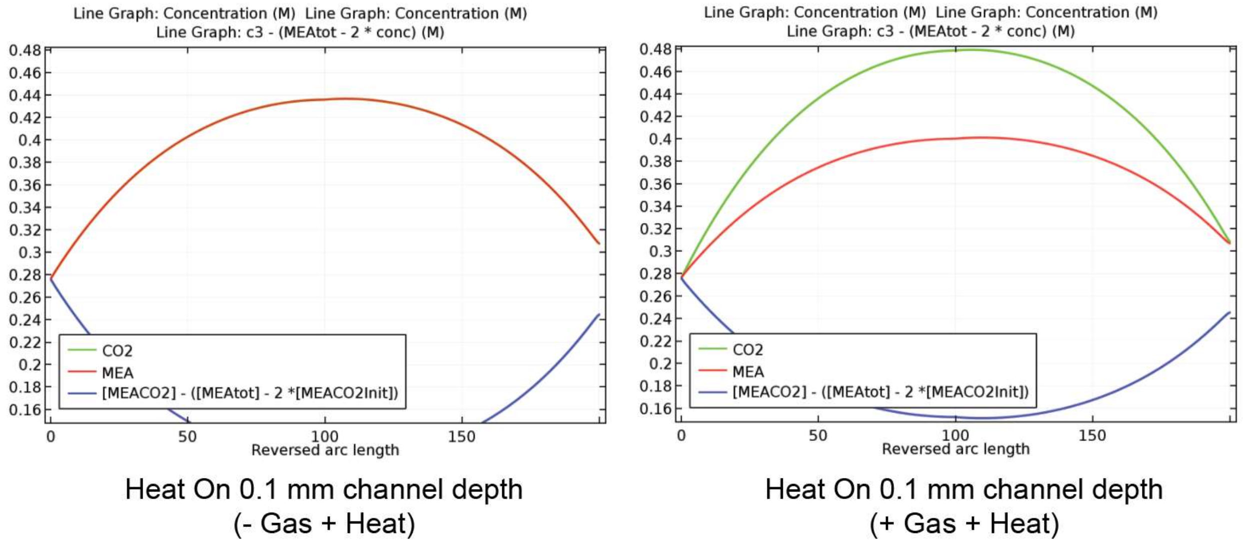


Supplementary Figure 19: Component concentrations along the PDMS adjacent face of the inlet and outlet channels (0.1 mm channel depth). Gas diffusion between channels disabled (left) and gas diffusion enabled (right). The concentration of MEA−$CO_2$ was graphed as ([MEA−$CO_2$] − $[MEA]_t$ − 2 ∗ $[MEA]_e$) where $[MEA]_t$ is the total concentration of MEA species in solution and $[MEA]_e$ is the equilibrium MEA concentration without applied heat.

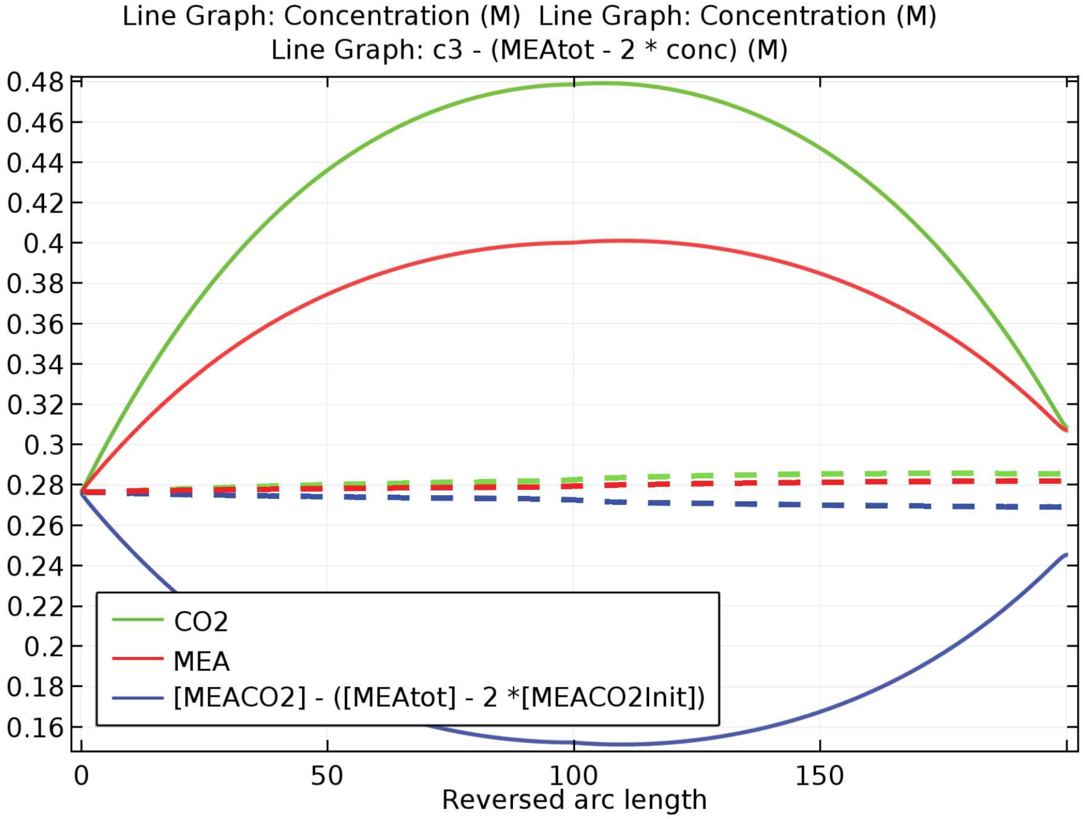


Supplementary Figure 20: Overlay of the computed concentrations in 1 mm (dashed lines) and 0.1 mm (solid lines) channel depth iterations of the (+ Gas, + Heat) countercurrent amplifier.

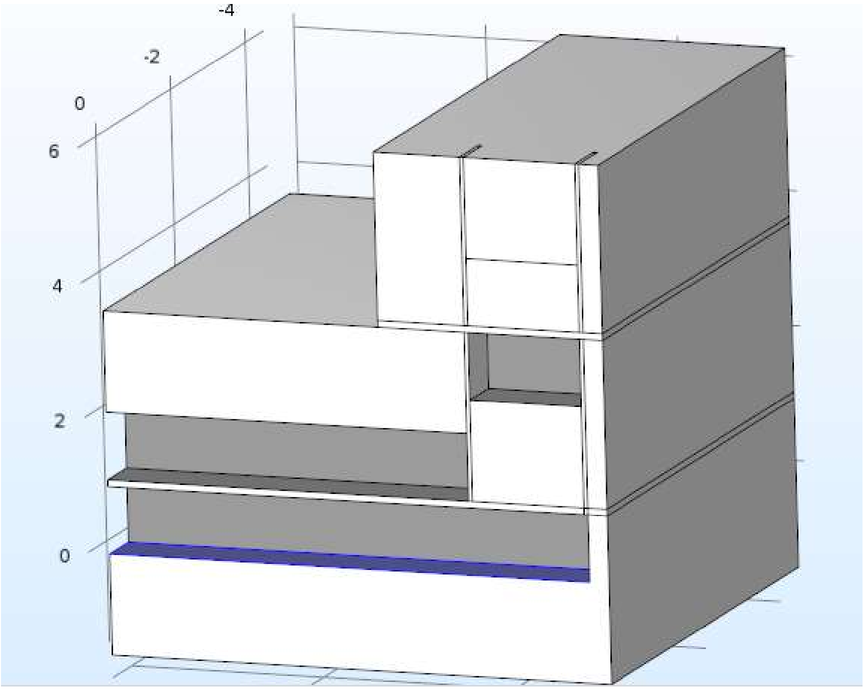


Supplementary Figure 21: COMSOL model of the experimental countercurrent amplifier. The highlighted face is the closest face to the heater in the outgoing capture fluid channel. The dimension along the length of the channel is not to scale for clarity.

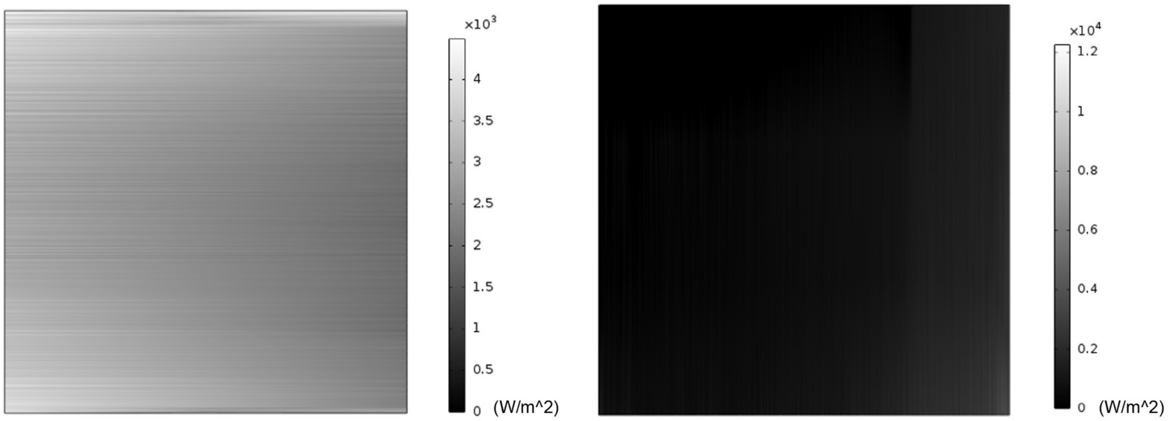


Supplementary Figure 22: Left – color map (W/m$^2$) of the face of the capture channel closest to the heater. Right – color map (W/m$^2$) of the face of the capture channel furthest from the heater.

## Supplementary Table 1: Distribution of raw CB particle sizes from ImageJ in PFO

| | Area (μm$^2$) |
|---|---|
| Mean | 812 |
| Stdv | 9785 |
| Min | 4 |
| Max | 265563 |

Supplementary Table 2: Distribution of perfluorinated CB particle sizes from ImageJ in PFO

| | Area ($\mu m^2$) |
|---|---|
| Mean | 21 |
| Stdv | 57 |
| Min | 4 |
| Max | 1501 |

Supplementary Table 3: Dimensions of the acrylic swim bladder system

| Part | Dimension (mm) |
|---|---|
| Acrylic Layer Thickness | 5.0 |
| Channel depth | 1.0 -or- 0.1 |
| Channel length | 100.0 |
| Membrane thickness | 0.140 |

Supplementary Table 4: Notable diffusion coefficients in MEA/H2O system. *Diffusion data could not be found for the MEA-CO2 species, so the value was assumed to be the same as for MEA.

| | Diffusion Coefficient $\left(10^{-5} \cdot \frac{cm^2}{sec}\right)$ |
|---|---|
| $CO_2$ in $MEA/H_2O$[3] | $6.955 * 10^{-8} \cdot e^{-\frac{1113}{T}}$ |
| $CO_2$ in PDMS[4] | 2.2 |
| MEA in $MEA/H_2O$[5] | $2.487 * 10^{-6} \cdot e^{-\frac{2436}{T}}$ |
| $MEA\text{-}CO_2$ in $MEA/H_2O$[*] | $2.487 * 10^{-6} \cdot e^{-\frac{2436}{T}}$ |

**Supplementary Note 1: Design of countercurrent device**

All countercurrent devices were machined from 5 mm thick acrylic sheets purchased from McMaster Carr. Fluid channels are all 1 mm wide by 1 mm deep square cross sections for ease of fabrication. Technical drawings of the channel layouts can be found in Supplementary Figures 1-3.

**Supplementary Note 2: PDMS/aluminum foil hybrid membrane**

A hybrid membrane was designed and fabricated as illustrated in Supplementary Figure 5 consisting of three layers. The two outer layers of PDMS maintain a consistent thermal resistivity compared to the original PDMS membrane as well as preventing corrosion of the aluminum layer by the MEA solution. The aluminum foil serves as a gas impermeable layer. Calculations detailing the thermal conductivity of the hybrid membrane compared to the PDMS membrane can be found below.

The thermal resistivities of the the hybrid and the original membrane were compared as follows

$$R_h = R_{PDMS} + R_{Al}$$

where $R\_h$, $R_{PDMS}$, and $R_{Al}$ are the thermal conductivities of the hybrid membrane, the two PDMS layers, and the aluminum foil layer respectively. $R_{PDMS}$ and $R_{Al}$ can be described by

$$R_{PDMS} = \frac{x_{PDMS}}{k_{PDMS}}$$

and

$$R_{Al} = \frac{x_{Al}}{k_{Al}}$$

Where $k_{PDMS}\,(0.15\,W \cdot m^{-1} \cdot K^{-1})$, $k_{Al}\,(237\,W \cdot m^{-1} \cdot K^{-1})$, and $x_{PDMS}$, $x_{Al}$ are the thermal conductivities and total layer thicknesses for PDMS and aluminum respectively.[1,2] Plugging the above values into equation 3.1 results in

$$R_h = \frac{0.000140\,m}{0.15\,\frac{W}{mK}} + \frac{0.000040\,m}{237\frac{W}{mK}} = 0.0009335\frac{W}{K}$$

This results in a 0.02 % difference in thermal resistance between the value of $0.0009333\frac{W}{K}$ for the 140 µm PDMS only membrane.

**Supplementary Note 3: Model parameters and reaction kinetics**

A simplified geometry was used in the model to reduce the computational load, enabling quicker iteration in model development. Pertinent values and dimensions can be found in Supplemental Tables 3-4.The relevant reaction can be seen in Supplementary Figure 15.

The reaction kinetics were obtained from work by Maeder et. al.[6] The forward and reverse rates are given by:

$$k_1 = \frac{k_B \cdot T}{h} \cdot e^{\frac{-38000\left(\frac{\mathrm{J}}{\mathrm{mol}}\right)}{R \cdot T}} \cdot e^{\frac{-47\left(\frac{J}{mol}\right)}{R}}$$

and

$$k_{-1} = \frac{k_B \cdot T}{h} \cdot e^{\frac{-61000\left(\frac{\mathrm{J}}{\mathrm{mol}}\right)}{R \cdot T}} \cdot e^{\frac{-4\left(\frac{\mathrm{J}}{\mathrm{mol}}\right)}{R}}$$

where $k_B$ is the Boltzmann constant, R is the gas constant (8.314 $JK^{-1}mol^{-1}$), h is the Plank constant, and T is the temperature in Kelvin.

The overall rate is then given by:

$$Rate = k_{-1}[MEA - CO_2] - k_1[CO_2][MEA]$$

or

$$Rate = \frac{k_B \cdot T}{h} \cdot \left( e^{\frac{-61000\left(\frac{\mathrm{J}}{\mathrm{mol}}\right)}{R \cdot T}} \cdot e^{\frac{-4\left(\frac{\mathrm{J}}{\mathrm{mol}}\right)}{R}} \cdot [MEA - CO_2] - e^{\frac{-3800\left(\frac{\mathrm{J}}{\mathrm{mol}}\right)}{R \cdot T}} \cdot e^{\frac{-47\left(\frac{\mathrm{J}}{\mathrm{mol}}\right)}{R}} \cdot [CO_2][MEA] \right)$$

The final rate is entered as shown for the formation rate of MEA and $CO_2$ and the reverse rate is entered for the formation of MEA-$CO_2$.

The initial concentrations for the reaction components can be found as shown below.

Given

$$K = \frac{k_1}{k_{-1}}$$

and

$$K = \frac{[CO_2][MEA]}{[MEA - CO_2]}$$

$[CO_2]$ can be solved for.

Assuming that $[CO_2] = [MEA]$ at equilibrium and given an initial MEA concentration ($[MEA_{tot}]$) of 5 M, the equilibrium can be written as

$$K = \frac{[CO_2]^2}{[MEA_{tot}] - [CO_2]}$$

which can be solved for $[CO_2]$.

$$[CO_2] = \frac{-1 + \sqrt{1 + 4 \cdot [MEAtot] \cdot K}}{2 \cdot K}$$

This can be approximated within the temperature range of interest by

$$[CO_2] \approx 0.00483 \left(\frac{M}{K}\right) \cdot T - 1.2(M)$$

The two previous expressions can be seen plotted Supplementary Figure 16.

Solving the for [$CO_2$] at the initial temperature of our system gives [$CO_2$] = 0.275 M

The above value was used as a starting point, but a concentration of 0.2762 M was found to be optimal in terms of reducing concentration swings at room temperature.

**Supplementary References**


1. Kuo, a C. M. Poly (dimethylsiloxane). in *Polymer Data Handbook* 411 (Oxford University Press, 1999).
2. Hatch, J. E. Aluminum Properties and Physical Metallurgy. *ASM ,Ohio, US* 424 (1984). doi:10.1361/appm1984p001
3. Samanta, A., Roy, S. & Bandyopadhyay, S. S. Physical solubility and diffusivity of N2O and CO2 in aqueous solutions of piperazine and (N-methyldiethanolamine + piperazine). *J. Chem. Eng. Data* **52,** 1381–1385 (2007).
4. Merkel, T. C., Bondar, V. I., Nagai, K., Freeman, B. D. & Pinnau, I. Gas sorption, diffusion, and permeation in poly(dimethylsiloxane). *J. Polym. Sci. Part B Polym. Phys.* **38,** 415–434 (2000).
5. Snijder, E. D., te Riele, M. J. M., Versteeg, G. F. & van Swaaij, W. P. M. Diffusion coefficients of several aqueous alkanolamine solutions. *J. Chem. Eng. Data* **38,** 475–480 (1993).
6. Conway, W. *et al.* Comprehensive Kinetic and Thermodynamic Study of the Reactions of CO2 (aq) and HCO3 – with Monoethanolamine (MEA) in Aqueous Solution. *J. Phys. Chem. A* **115,** 14340–14349 (2011).
